# The mitochondria-targeted antioxidant SkQ1 but not N-acetylcysteine reverses aging-related biomarkers in rats

**DOI:** 10.18632/aging.100493

**Published:** 2012-10-28

**Authors:** Nataliya G. Kolosova, Natalia A. Stefanova, Natalia A. Muraleva, Vladimir P. Skulachev

**Affiliations:** ^1^ Institute of Cytology and Genetics SB RAS, Novosibirsk, Russia; ^2^ Institute of Mitoengineering, Moscow, Russia; ^3^ Lomonosov Moscow State University, Belozersky Institute of Physico-Chemical Biology, Moscow, Russia

**Keywords:** aging, SkQ1, NAC, growth hormone, IGF-1, testosterone

## Abstract

Although antioxidants have been repeatedly tested in animal models and clinical studies, there is no evidence that antioxidants reduce already developed age-related decline. Recently we demonstrated that mitochondria-targeted antioxidant 10-(6'-plastoquinonyl) decyltriphenylphosphonium (SkQ1) delayed some manifestations of aging. Here we compared effects of SkQ1 and N-acetyl-L-cysteine (NAC) on age-dependent decline in blood levels of leukocytes, growth hormone (GH), insulin-like growth factor-1 (IGF-1), testosterone, dehydroepiandrosterone (DHEA) in Wistar and senescence-accelerated OXYS rats. When started late in life, supplementation with SkQ1 not only prevented age-related decline but also significantly reversed it. With NAC, all the observed effects were of the lower magnitude compared with SkQ1 (in spite of that dose of NAC was 16000 times higher). We suggest that supplementation with low doses of SkQ1 is a promising intervention to achieve a healthy ageing.

## INTRODUCTION

Aging is commonly defined as progressive deleterious alterations that lead to increased risk of disease and death with advancing age [1-3]. Several potential therapeutic approaches are now available to slow down the age-related functional decline of the organism [4-17] including treatment with antioxidants [1, 18, 19]. Indeed, generation of reactive oxygen species (ROS) by mitochondria is considered as one of mechanisms of aging [20-28]. However, current antioxidants are not selective to mitochondria and that might hamper their effectiveness [29]. Over the course of seven years we investigated retardation of aging with a mitochondria-targeted antioxidant SkQ1 [10-(6'-plastoquinonyl) decyltriphenylphosphonium]. SkQ1 is an antioxidant selectively targeted to mitochondria that protects mitochondria from oxidative damage and which has been shown to decrease mitochondrial damage in animal models of oxidative stress [29, 30]. We have shown that SkQ1 increased the median lifespan of organisms and also delayed, arrested, and in some cases even reversed development of many age-related pathological traits [29-34].

A complex change of the immune system occurs with aging. Immuno-senescence is defined as decreased cellular reactivity, and imbalance between inflammatory and anti-inflammatory networks, which results in low-grade, chronic, pro-inflammatory condition also known as “inflammaging” [35-39]. Aging affects all immune cells, and it leads to high susceptibility to infections and increased mortality observed in the elderly. Therefore age-related changes in immune cells could serve as a marker of health, biological age and longevity [40].

Here we evaluated the effect of the mitochondria-targeted antioxidant SkQ1 on markers of aging in the old OXYS rats, a unique animal model of accelerated senescence and age-related diseases, as well as normal Wistar rats. OXYS rats spontaneously develop several pathological phenotypes similar to human geriatric disorders including cataract, retinopathy, osteoporosis, high blood pressure and behavioral alterations [41-50]. Most of these manifestations of accelerated aging develop in OXYS rats between 1 and 3 months of life. Recently we showed that SkQ1 at nanomolar concentrations is capable not only to prevent decline of the immune system and development of cataract and retinopathy but also can reverse already developed pathological changes in the lens and retina of OXYS rats as well as some age-related alterations in behavior [29, 51-53].

For comparison we used N-acetyl-L-cysteine (NAC) as a non-targeted antioxidant. NAC has been shown to prevent age-related cognitive defects and oxidative decline of mitochondrial functions in the brain [54, 55].

## RESULTS

### Body weight

The body weight of 19-month-old rats was measured before the treatment with antioxidants. Rats were randomly assigned to control and experimental groups. OXYS rats are characterized by lower body weight in comparison with Wistar rats. Accordingly, before treatment at the age of 19 months the body weight was dependent only on the genotype (F_1.82_ = 472.7, p < 0.000) and was lower in OXYS rats (Table 1). At the start of treatment with antioxidants experimental groups did not differ in weight (p = 0.34 for Wistar, p = 0.52 for OXYS). At the end of the 4-month treatment with NAC and SkQ1 the body weight remained lower in OXYS rats (F_1.82_ = 245.6, p < 0.000) and it was not affected by the antioxidants (F_2.82_ = 1.6, p = 0.2). A paired dependent comparison showed that body weight of OXYS rats treated with NAC at the age of 23 months was even lower than their weight at the age of 19 months (p < 0.015). OXYS rats treated with SkQ1 showed tendency to lose weight, whereas the body weight of control OXYS rats was not changed by the age of 23 months.

**Table 1:** Body weight (g) of control and SkQ1-or NAC-treated Wistar and OXYS rats at the age of 19 and 23 months

Strain	Wistar			OXYS		
Treatment	0	SkQ1	NAC	0	SkQ1	NAC
19 months	612±18*	630±18*	637±17*	404±9*	424±14*	411±6*
23 months	560±27*	642±20*	604±27*	393±8*	390±8*	385±9^*

* statistically significant differences between the strains of the same age;

^ statistically significant differences between 19- and 23-month-old rats (paired-dependent comparisons of the same animals).

### The white blood cell analyses

In agreement with earlier findings [56], we observed an age-dependent decrease in the number of lymphocytes and an increase in neutrophils (Tabl. 2). The counts of neutrophils in the peripheral blood of Wistar rats at the age of 19 months were higher than those in 3 months old rats (p < 0.05, comparison of group mean values); and number of neutrophils in 23 months old animals was higher than that in 19 months old rats (p < 0.009, paired dependent comparisons of the same animals). At the same time the counts of lymphocytes in the blood of Wistar rats decreased (p < 0.009 for 19 months old and p < 0.04 for 23 months old). In OXYS rats, these parameters also varied with age, but the increase in the counts of neutrophils and decrease in lymphocytes were statistically significant only at the age of 19 months compared with the 3-month-old animals (p < 0.05). Treatment of Wistar rats with NAC almost completely prevented the drop in lymphocytes/neutrophils ratio, while 4 months treatment with SkQ1 not only prevented its decrease, but actually increased this ratio almost to the level of young rats. In OXYS rats, neither aging from 19 to 23 months nor antioxidants significantly affected the white blood cell counts.

**Table 2:** White blood cell counts in Wistar and OXYS rats at the age of 3, 19 and 23 months. Effects of 4 months treatment with SkQ1 or NAC. The results are given as % of the total number of white blood cells

Strain	Age, month	Treatment	Lymphocytes	Neutrophils	Eosinophils	Monocytes	Lymphocytes/Neutro phils ratio
Wistar	3	0	74.1±5.8	20.4±0.21	2.13±0.25	4.10±0.25	3.63±0.41
	19	0	63.0±1.32^	29.4±1.27^	3.36±0.29^	3.43±0.20	2.46±0.14^
	23	0	54.5±4.42^	38.6±4.47^	4.50±1.26	2.38±0.34^	1.77±0.29^
		SkQ1	66.9±3.19#	25.9±2.92#	4.00±0.83	3.22±0.28	2.92±0.39#
		NAC	62.4±3.72	31.7±3.79	2.93±0.66	3.00±0.33	2.26±0.34
OXYS	3	0	73.1±6.1	19.36±1.6	2.22±0.21	5.27±0.52	3.80±0.35
	19	0	66.0±0.91^	28.0±0.82^	2.77±0.20	3.0±0.21^	2.54±0.10^
	23	0	64.3±1.78*	30.3±1.91	2.53±0.45*	2.87±0.31	2.28±0.19
		SkQ1	57.1±3.17	38.4±3.05#	1.83±0.53#	2.65±0.31	1.76±0.22
		NAC	58.3±2.14	36.6±2.23	2.23±0.28	2.86±0.18	1.77±0.15

* statistically significant difference between the strains of the same age;

# significant effect of the drug within the strain;

^ significant age-related differences from the previous age within the strain.

### Serum GH levels

The level of GH naturally was decreased with age in rats of both strains albeit more profoundly in OXYS rats than in Wistar rats (p < 0.0001 for age of 3 months, p < 0.009 for 19 months and p < 0.008 for 23 months). Before the treatment with antioxidants GH levels depended only on the genotype (F_1.42_ = 60.96, p < 0.000) and at the age of 19 months OXYS rats had lower levels of GH (Table 3). At the start of supplementation with antioxidants the experimental groups did not differ in the levels of GH (p = 0.49 for Wistar, p = 0.52 for OXYS).

**Table 3:** Levels of GH, IGF-1, DHEA-S and testosterone (ng/ml) in serum of intact Wistar and OXYS rats at the age of 3, 19 and 23 months and of rats treated with SkQ1 or NAC from the age of 19 months to 23 months

Strain	Age, month	Treatment	GH	IGF-1	Testosterone	DHEA
Wistar	3	0	8.14±0.2	535±62	1.42±0.3	-
	19	0	5.28±0.1+	483±7	0.61±0.04+	0.66±0.01
	23	0	4.98±0.2+	448±12+	0.60±0.07	0.63±0.02+
		SkQ1	5.50±0.1 ^#^^	500 ± 9^#^	0.61±0.05	0.66±0.01
		NAC	5.37±0.2^	486±10^#^	0.61±0.03	0.66±0.03
OXYS	3	0	6.24±0.3*	498±64	1.99±0.31	-
	19	0	4.57±0.1 *+	444±7*+	0.64±0.06+	0.58±0.01*
	23	0	4.27±0.1*+	399±9*+	0.56±0.02	0.55±0.03*+
		SkQ1	4.80±0.1^#^^	499±9 ^#^^	0.58±0.02	0.60±0.02^
		NAC	4.58±0.08^	449±13^#^	0.57±0.02	0.59±0.02

* statistically-significant difference between the strains of the same age;

# significant effect of the drug within the strain;

+ significant age-related differences from the previous age within the strain,

^ statistically-significant difference between the levels before and after treatment with SkQ1 or NAC (paired-dependent comparisons of the same animals).

After 4-month of treatment with NAC or SkQ1 GH levels remained lower in OXYS rats (F_1.42_ = 49.6, p < 0.000) and were affected by antioxidants (F_2.42_ = 8.8, p < 0.0008). In both Wistar and OXYS rats treated with SkQ1, GH levels were significantly higher than in control groups of the corresponding strain (p < 0.004 and p <0.02, respectively). In Wistar rats treated with NAC - no difference was found. Yet a paired dependent comparison showed that GH levels in Wistar rats treated with SkQ1 and NAC were higher at the age of 23 months than at the age of 19 months (p < 0.009 and p < 0.046, respectively). A paired dependent comparison showed a small but significant increase in GH levels from the age of 19 months to 23 months in OXYS rats treated with SkQ1 (p < 0.000) or NAC (p < 0.046). In control OXYS rats GH levels decreased significantly (p < 0.02) by the age of 23 months. In addition, SkQ1 treated OXYS rats and control Wistar rats of the same age (23 months) did not differ in GH levels.

### Serum IGF-I levels

At the age of 3 months, serum IGF-1 levels were maximal in Wistar and OXYS rats and there was no difference in IGF-1 levels between the strains. IGF-1 levels decreased in rats with age, more profoundly in OXYS rats than in Wistar rats (p < 0.002 for the age of 19 months and p < 0.048 for 23 months). Before treatment with antioxidants IGF-1 levels were dependent on the genotype (F_1.42_ = 12.3, p < 0.002) and at the age of 19 months IGF-1 was lower in OXYS rats than in Wistar rats (Table 3). At the start of treatment with antioxidants experimental groups did not differ in the levels of IGF-1 (p = 0.87 for Wistar and p = 0.81 for OXYS rats).

After 4 months treatment with antioxidants IGF-1 levels were dependent on the genotype, (F_1.42_ = 8.1, p < 0.008) and were affected by antioxidants (F_2.42_ = 19.4, p < 0.00). In Wistar rats treated with either SkQ1 or NAC, IGF-1 levels were significantly higher (p < 0.0002 and p < 0.014, respectively) than in the control group. The paired dependent comparison showed that IGF-1 levels in 23-month-old Wistar rats treated with either NAC or SkQ1 were even higher than they have been at the age of 19 months (p = 0.032), while in the control rats IGF-1 was significantly lower (p < 0.001).

From the age of 19 to 23 months IGF-1 levels in control group of OXYS rats also decreased significantly (p < 0.001). In OXYS rats treated with either SkQ1 or NAC, IGF-1 levels were significantly higher than in the control group (p < 0.013 and p < 0.031, respectively). The paired dependent comparison showed that in rats treated with NAC, at the age of 23 months IGF-1 levels remained similar to the 19-month-old animals, whereas, in the rats treated with SkQ1 its level increased significantly (p < 0.000). In addition, in 23-month-old OXYS rats treated with NAC, IGF-1 levels were similar to those in Wistar control rats of the same age, but in OXYS rats treated with SkQ1, IGF-1 levels were similar to IGF-1 levels of young Wistar rats.

### Serum testosterone and DHEA levels

ANOVA analyses showed that the level of testosterone in Wistar and OXYS rats was maximal at the age of 3 months and decreased by the age of 19 months in both strains (F_2.62_ = 74.7, p < 0.00) and remained unchanged by the age of 23 months. Treatment with NAC and SkQ1 had no effect on the testosterone level (Table 3).

We did not measure DHEA in three-month old animals. At the age of 19 months, DHEA level was slightly lower in OXYS rats (F_1.42_ = 19.2, p < 0.0001). In 23 months old Wistar and OXYS rats, level of DHEA differs only slightly from that in 19 months old animals (Table 3).

## DISCUSSION

Our results indicate that when started late in life, treatment with SkQ1 not only prevented age-related decline, but also partially reversed it. Effects of NAC were of the lower magnitude compared to SkQ1, despite the higher dose of NAC used.

One reason for body weight loss in old age is sarcopenia - a gradual decline in muscle mass. After 4 months treatment with antioxidants the body weight of OXYS and Wistar rats decreased only slightly. In SkQ1 treated group weight was very similar to weight of control rats in both rat strains. These findings are consistent with unpublished data of L.E. Bakeeva and V.B. Saprunova, who found that SkQ1 reduced the age-related decline in muscle mass in OXYS and Wistar rats. Another reason for body weight reduction in old age is osteoporosis, which results in increased risk of fractures. Feeding SkQ1 prevented loss of mineral content associated with senile osteoporosis in OXYS rats [34]. In our study the paired dependent comparison showed that body weight decreased significantly (p < 0.015) only in OXYS rats treated with NAC.

White blood cells (WBC) counts could serve as a marker of health, biological age and longevity. In humans, lymphocytes increase early in life until age of 16–21 years [56]. Some studies indicate that number of lymphocytes decreases with age [57-59]. In line with these findings, we observed that the lymphocyte/neutrophil ratio was high in the 3-month-old Wistar and OXYS rats and decreased with age: the counts of lymphocytes were decreased and neutrophils were increased. Lymphocytes are important effector cells and therefore their activation is essential for immune responses [57]. Diminished lymphocyte production and function are major contributors to disease in elderly [56]. 4 months treatment with NAC almost completely prevented the decrease in ratio between lymphocytes and neutrophils in 23-month-old Wistar rats compared with 19-month-old ones. SkQ1 increased the lymphocyte/neutrophil ratio, and thereby partially reversed decline of this parameter, which was observed at the age of 19 months. Our present results in Wistar rats are in line with the previous reports that NAC [60, 61] and SkQ1 [32,58] prevent the age-linked decrease in lymphocyte level in mice. However, both antioxidants did not affect the blood lymphocyte/neutrophil ratio in OXYS rats. The age-related decrease in lymphocytes is a consequence of involution of thymus, the major organ of lymphocyte maturation. The OXYS rats exhibit accelerated involution of the thymus and SkQ1 reduces age-related thymic involution in both OXYS and normal Wistar rats [52]. It is possible that lack of SkQ1 and NAC effects on the WBC count in old OXYS rats is associated with impairment in bone marrow hematopoiesis. We have previously reported the age-associated changes in the functional status of hematopoietic stem cells in OXYS rats [62]. It can be assumed that in OXYS rats the lymphocyte/neutrophil ratio was already stabilized at low level in the 19 month-old rats so that antioxidants could not have a favorable effect. Antioxidants had no effect on the levels of eosinophils and monocytes in both rat strains.

Circulating GH levels are at the highest during the neonatal period; they decrease during childhood, peak again during puberty and fall dramatically in the elderly [63]. A reduction in GH level in older humans and rodents correlates with a decline in serum levels of an anabolic mediator IGF-1 [64]. The present study confirmed an age-dependent GH and IGF-1 decrease in rats of both strains. In addition, we showed that the serum GH level in all studied groups of OXYS rats was lower than in age-matched Wistar rats. Interstrain differences were highest in the three-month old animals (23%), while at the age of 19 and 23 months difference was 13% and 14%, respectively. There were no interstrain differences in the blood levels of IGF-1 in the 3-month-old animals but at the ages of 19 and 23 months they were slightly (but statistically significant) reduced in OXYS rats (by 9 and 11%, respectively).

Our study also showed for the first time that NAC supplementation from the age 19 to 23 months fully prevented the GH and IGF-I decline in both Wistar and OXYS rats. SkQ1 not only stopped the decline in hormone levels between the ages of 19 and 23 months, but it also increased the levels of GH and IGF-I above the levels of those found in 19 month-old animals (Table 3). It is well known that GH/IGF-I plays an important role in brain aging [65, 66]. Age-related reduction in the activities of somatotropic axis may influence brain function in the elderly [67]. Recently we have shown that SkQ1 treatment of the middle-aged (12 month) Wistar and OXYS rats had beneficial effects on the locomotor and exploratory activity. SkQ1 also decreased anxiety compared to age-matched controls as well as significantly improved visual ability of the OXYS rats, which suffered from retinopathy and cataract [49]. In the present study, we observed a positive effect of SkQ1 and NAC on the behavior of rats of both strains (data are not shown). In the last series of experiments, we studied effect of SkQ1 on the levels of growth hormone and IGF-1 in 3-month-old rats (data are not shown). We found that SkQ1 caused small (about 25%) but statistically valid increase in the blood hormone level. In addition, SkQ1 prevented the development of retinopathy and cataract and had beneficial effects on behavior, learning ability and memory of OXYS rats. We suggest that the recovery of GH - IGF-I in old age to the levels of those in young age can have a positive impact on the function of the aging brain and the immune system.

At the age between 3 and 19 months, the testosterone levels fell and then remained unchanged between 19 and 23 months in rats of both strains. It was not surprising that there was no interstrain difference in the testosterone level even in old animals. As was previously shown in our group, OXYS males demonstrate an early decrease in sexual motivation; however a decrease in hormonal component of sexual behavior was not detected in aged OXYS males [68]. Recently we showed that SkQ1 is effective not only in preventing but also in reducing already developed age-related decline in male sexual behavior [69]. In the present study, we did not evaluate the sexual behavior, and neither SkQ1 nor NAC treatments affected testosterone levels in period between 19 and 23 months (Table 3). Noteworthy, NAC partially inhibits the mTOR (Target of Rapamycin) pathway [70]. Given the involvement of mTOR in cellular and organismal aging as well as age-related diseases [71-78], slight inhibition of mTOR may contribute to the therapeutic effects of this non-selective antioxidant.

DHEA and its sulfate-bound form (DHEAS) are important precursors of sex steroid hormones. Structure of DHEA is similar to testosterone and levels of both hormones reach their maximal levels in puberty and decrease dramatically with age [79]. Given its multiple metabolic effects, this decline in DHEA levels has been thought to play a role in the aging process [80]. In this study, we can assume that the DHEA levels are maximal in 3-month-old Wistar and OXYS rats. At the age between 19 and 23 months, DHEA levels decreased and SkQ1 increased DHEA only slightly (by 5%).

Deficiencies in multiple hormones are a biomarker of health status in older persons [79].

Here we conclude that SkQ1 not only prevented age-associated hormonal alterations but partially reversed them. These results suggest that supplementation with low doses of SkQ1, even in chronologically and biologically aged subjects seem to be a promising strategy to maintain health and retard the aging process.

## MATERIALS AND METHODS

### Animals and diet

Male senescence-accelerated OXYS and age-matched male Wistar rats were obtained from the Breeding Experimental Animal Laboratory of the Institute of Cytology and Genetics (ICG), Siberian Division of the Russian Academy of Sciences (Novosibirsk, Russia). All the experiments on rats were carried out according to Animal Care Regulations of ICG Institute of Cytology and Genetics, Novosibirsk. The OXYS rat strain was established based on Wistar rat strain at the Institute of Cytology and Genetics as described earlier [51, 52] and registered in the Rat Genome Database (http://rgd.mcw.edu/). At the age of 4 weeks, the pups were taken away from their mothers and housed in groups of five animals per cage (57×36×20 cm) and kept under standard laboratory conditions (at 22±2°C, 60% relative humidity, and natural light), provided with a standard rodent feed, PK-120-1, Ltd. (Laboratorsnab, Russia), and given water ad libitum.

Starting from the age of 19 months OXYS and Wistar rats were randomly assigned to three groups (n = 17–24): control diet, diet supplemented with 250 nmol SkQ1 (synthesized as described earlier [33]) or 650 mg NAC (MP Biomedicals, LLC, France) per kg of body weight per day. The weight was measured before the start of treatment and at the end of experiment.

### Hormone levels and the white blood cell counts

The levels of GH, IGF-1, testosterone and DHEA-S in the blood serum were analyzed before and after treatment with SkQ1 or NAC in OXYS and Wistar rats (two times total for each rat) and compared with intact 3-month-old rats (n=10) of the same strains. GH, DHEA were measured by ELISA (Creative Diagnostics, USA). IGF-1 was measured by ELISA (ALPCO Diagnostics, Salem, NH). Testosterone was measured by ELISA (JSC Vector-Best, Russia). The assays were run using the manufacturer's instructions. For WBC counts peripheral blood was drawn from the tail vein. Blood smears were fixed in methanol and subsequently stained with Wright-Giemsa.

### Statistical analysis

The data were analyzed using repeated measures ANOVA and nonparametric tests with the statistical package Statistica 6.0. Two-way ANOVA was used to evaluate the differences between rat strains (genotypes) and effects of treatment (antioxidants). To validate the effect of the diets on parameters, the genotype and antioxidants were chosen as independent variables. A Newman-Keuls *post hoc* test was applied to significant main effects and interactions in order to estimate the differences between particular sets of means. One-way ANOVA was used for individual group comparisons. Data are represented as mean ± S.E.M. Comparisons between means were analyzed with one-way or repeated measures analysis of variance (ANOVA). Results were considered statistically significant if p value was less than 0.05.

## References

[R1] Harman D (1956). Aging: a theory based on free radical and radiation chemistry. J Gerontol.

[R2] Harman D (2003). The free radical theory of aging. Antioxid Redox Signal.

[R3] Skulachev VP (2011). Aging as a particular case of phenoptosis, the programmed death of an organism (a response to Kirkwood and Melov “On the programmed/non-programmed nature of ageing within the life history”). Aging (Albany NY).

[R4] Anisimov VN, Piskunova TS, Popovich IG, Zabezhinski MA, Tyndyk ML, Egormin PA, Yurova MV, Rosenfeld SV, Semenchenko AV, Kovalenko IG, Poroshina TE, Berstein LM (2010). Gender differences in metformin effect on aging, life span and spontaneous tumorigenesis in 129/Sv mice. Aging (Albany NY).

[R5] Anisimov VN, Berstein LM, Popovich IG, Zabezhinski MA, Egormin PA, Piskunova TS, Semenchenko AV, Tyndyk ML, Yurova MN, Kovalenko IG, Poroshina TE (2011). If started early in life, metformin treatment increases life span and postpones tumors in female SHR mice. Aging (Albany NY).

[R6] Anisimov VN, Zabezhinski MA, Popovich IG, Piskunova TS, Semenchenko AV, Tyndyk ML, Yurova MN, Rosenfeld SV, Blagosklonny MV (2011). Rapamycin increases lifespan and inhibits spontaneous tumorigenesis in inbred female mice. Cell Cycle.

[R7] Baur JA, Sinclair DA (2006). Therapeutic potential of resveratrol: the in vivo evidence. Nat Rev Drug Discov.

[R8] Burks TN, Cohn RD (2011). One size may not fit all: anti-aging therapies and sarcopenia. Aging (Albany NY).

[R9] Chateau MT, Araiz C, Descamps S, Galas S (2010). Klotho interferes with a novel FGF-signalling pathway and insulin/Igf-like signalling to improve longevity and stress resistance in Caenorhabditis elegans. Aging (Albany NY).

[R10] Goldberg AA, Richard VR, Kyryakov P, Bourque SD, Beach A, Burstein MT, Glebov A, Koupaki O, Boukh-Viner T, Gregg C, Juneau M, English AM, Thomas DY, Titorenko VI (2010). Chemical genetic screen identifies lithocholic acid as an anti-aging compound that extends yeast chronological life span in a TOR-independent manner, by modulating housekeeping longevity assurance processes. Aging (Albany NY).

[R11] Guarente L (2007). Sirtuins in aging and disease. Cold Spring Harb Symp Quant Biol.

[R12] Hofseth LJ, Singh UP, Singh NP, Nagarkatti M, Nagarkatti PS (2010). Taming the beast within: resveratrol suppresses colitis and prevents colon cancer. Aging (Albany NY).

[R13] Khanna A, Kapahi P (2011). Rapamycin: killing two birds with one stone. Aging (Albany NY).

[R14] Menendez JA, Cufi S, Oliveras-Ferraros C, Vellon L, Joven J, Vazquez-Martin A (2011). Gerosuppressant metformin: less is more. Aging (Albany NY).

[R15] Morselli E, Galluzzi L, Kepp O, Criollo A, Maiuri MC, Tavernarakis N, Madeo F, Kroemer G (2009). Autophagy mediates pharmacological lifespan extension by spermidine and resveratrol. Aging (Albany NY).

[R16] Moskalev A, Shaposhnikov M (2011). Pharmacological inhibition of NF-kappaB prolongs lifespan of Drosophila melanogaster. Aging (Albany NY).

[R17] Wood JG, Rogina B, Lavu S, Howitz K, Helfand SL, Tatar M, Sinclair D (2004). Sirtuin activators mimic caloric restriction and delay ageing in metazoans. Nature.

[R18] Rattan SIS (2005). Anti-ageing strategies: prevention or therapy. EMBO Rep.

[R19] Tong JJ, Schriner SE, McCleary D, Day BJ, Wallace DC (2007). Life extension through neurofibromin mitochondrial regulation and antioxidant therapy for neurofibromatosis-1 in Drosophila melanogaster. Nat Genet.

[R20] Dröge W, Schipper HM (2007). Oxidative stress and aberrant signaling in aging and cognitive decline. Aging Cell.

[R21] Guachalla LM, Rudolph KL (2010). ROS induced DNA damage and checkpoint responses: influences on aging?. Cell Cycle.

[R22] Hwang AB, Lee SJ (2011). Regulation of life span by mitochondrial respiration: the HIF-1 and ROS connection. Aging (Albany NY).

[R23] Lindner AB, Demarez A (2009). Protein aggregation as a paradigm of aging. Biochim Biophys Acta.

[R24] Pani G (2010). P66SHC and ageing: ROS and TOR?. Aging (Albany NY).

[R25] Papaconstantinou J, Hsieh CC (2010). Activation of senescence and aging characteristics by mitochondrially generated ROS: how are they linked?. Cell Cycle.

[R26] Sanz A, Fernandez-Ayala DJ, Stefanatos RK, Jacobs HT (2010). Mitochondrial ROS production correlates with, but does not directly regulate lifespan in Drosophila. Aging (Albany NY).

[R27] Vigneron A, Vousden KH (2010). p53, ROS and senescence in the control of aging. Aging (Albany NY).

[R28] Witte ME, Geurts JJ, de Vries HE, van der Valk P, van Horssen J (2010). Mitochondrial dysfunction: a potential link between neuroinflammation and neurodegeneration?. Mitochondrion.

[R29] Skulachev VP, Anisimov VN, Antonenko YN, Bakeeva LE, Chernyak BV, Erichev VP, Filenko OF, Kalinina NI, Kapelko VI, Kolosova NG, Kopnin BP, Korshunova GA, Lichinitser MR, Obukhova LA, Pasyukova EG, Pisarenko OI, Roginsky VA, Ruuge EK, Senin II, Severina II, Skulachev MV, Spivak IM, Tashlitsky VN, Tkachuk VA, Vyssokikh MY, Yaguzhinsky LS, Zorov DB (2009). An attempt to prevent senescence: a mitochondrial approach. Biochim Biophys Acta.

[R30] Skulachev MV, Antonenko YN, Anisimov VN, Chernyak BV, Cherepanov DA, Chistyakov VA, Egorov MV, Kolosova NG, Korshunova GA, Lyamzaev KG, Plotnikov EY, Roginsky VA, Savchenko AY, Severina II, Severin FF, Shkurat TP, Tashlitsky VN, Shidlovsky KM, Vyssokikh MY, Zamyatnin AA, Zorov DB, Skulachev VP (2011). Mitochondrial-targeted plastoquinone derivatives. Effect on senescence and acute age-related pathologies. Curr Drug Targets.

[R31] Anisimov VN, Bakeeva LE, Egormin PA, Filenko OF, Isakova EF, Manskikh VN, Mikhelson VM, Panteleeva AA, Pasyukova EG, Pilipenko DI, Piskunova TS, Popovich IG, Roshchina NV, Rybina OY, Saprunova VB, Samoylova TA, Semenchenko AV, Skulachev MV, Spivak IM, Tsybul'ko EA, Tyndyk ML, Vyssokikh MY, Yurova MN, Zabezhinsky MA, Skulachev VP (2008). Mitochondria-targeted plastoquinone derivatives as tools to interrupt execution of the aging program. 5. SkQ1 prolongs lifespan and prevents development of traits of senescence. Biochemistry (Mosc).

[R32] Anisimov VN, Egorov MV, Krasilshchikova MS, Lyamzaev KG, Manskikh VN, Moshkin MP, Novikov EA, Popovich IG, Rogovin KA, Shabalina IG, Shekarova ON, Skulachev MV, Titova TV, Vygodin VA, Vyssokikh MY, Yurova MN, Zabezhinsky MA, Skulachev VP (2011). Effects of the mitochondria-targeted antioxidant SkQ1 on lifespan of rodents. Aging (Albany NY).

[R33] Antonenko YuN, Avetisyan AV, Bakeeva LE, Chernyak BV, Chertkov VA, Domnina LV, Ivanova OYu, Izyumov DS, Khailova LS, Klishin SS, Korshunova GA, Lyamzaev KG (2008). Mitochondria-targeted plastoquinone derivatives as tools to interrupt execution of an aging program. 1. Cationic plastoquinone derivatives: synthesis and in vitro studies. Biochemistry (Moscow).

[R34] Neroev VV, Archipova MM, Bakeeva LE, Fursova AZh, Grigorian EN, Grishanova AY, Iomdina EN, Ivashchenko ZhN, Katargina LA, Khoroshilova-Maslova IP, Kilina OV, Kolosova NG, Kopenkin EP, Korshunov SS, Kovaleva NA, Novikova YP, Philippov PP, Pilipenko DI, Robustova OV, Saprunova VB, Senin II, Skulachev MV, Sotnikova LF, Stefanova NA, Tikhomirova NK, Tsapenko IV, Shchipanova AI, Zinovkin RA, Skulachev VP (2008). Mitochondriatargeted plastoquinone derivatives as tools to interrupt execution of the aging program. 4. Agerelated eye disease. SkQ1 returns vision to blind animals. Biochemistry (Moscow).

[R35] Franceschi C, Capri M, Monti D, Giunta S, Olivieri F, Sevini F, Panourgia MP, Laura Invidia L, Celani L, Scurti S, Cevenini E, Castellani GC, Salvioli S (2007). Inflammaging and anti-inflammaging: a systemic perspective on aging and longevity emerged from studies in humans. Mech. Ageing Dev.

[R36] Lisanti MP, Martinez-Outschoorn UE, Pavlides S, Whitaker-Menezes D, Pestell RG, Howell A, Sotgia F (2011). Accelerated aging in the tumor microenvironment: connecting aging, inflammation and cancer metabolism with personalized medicine. Cell Cycle.

[R37] Pavlides S, Tsirigos A, Vera I, Flomenberg N, Frank PG, Casimiro MC, Wang C, Pestell RG, Martinez-Outschoorn UE, Howell A, Sotgia F, Lisanti MP (2010). Transcriptional evidence for the “Reverse Warburg Effect” in human breast cancer tumor stroma and metastasis: similarities with oxidative stress, inflammation, Alzheimer's disease, and “Neuron-Glia Metabolic Coupling”. Aging (Albany NY).

[R38] Weiss EP, Villareal DT, Fontana L, Han DH, Holloszy JO (2011). Dehydroepiandrosterone (DHEA) replacement decreases insulin resistance and lowers inflammatory cytokines in aging humans. Aging (Albany NY).

[R39] Ye J, Keller JN (2010). Regulation of energy metabolism by inflammation: a feedback response in obesity and calorie restriction. Aging (Albany NY).

[R40] Wessels I, Jansen J, Rink L, Uciechowski P (2010). Immunosenescence of polymorphonuclear neutrophils. ScientificWorld Journal.

[R41] Agafonova IG, Rjtelnikov VN, Mishchenko NP, Kolosova NG (2010). Comparative study of the influence histochrome and mexidol on structural and functional characteristics of premature aging of the brain of rats OXYS by MRI. Bull Exp Biol Med.

[R42] Kolosova NG, Stefanova NA, Sergeeva SV, Gariépy Q, Ménard R (2009). OXYS rats: a prospective model for evaluation of antioxidant availability in prevention and therapy of accelerated aging and age-related cognitive decline: Handbook of Cognitive Aging: Causes, Proceses.

[R43] Kolosova NG, Akulov AE, Stefanova NA, Moshkin MP, Savelov AA, Koptyug IV, Panov AV, Vavilin VA (2011). Effect of malate on the development of rotenone-induced brain changes in Wistar and OXYS rats: An MRI study. Dokl Biol Sci.

[R44] Loskutova LV, Kolosova NG (2000). Emotional state and one trial learning in OXYS rats with hereditarily elevated production of oxygen radicals. Bull Exp Biol Med.

[R45] Loskutova LV, Zelenkina LM (2002). Impairment of latent inhibition in OXYS rats with hereditary syndrome of premature aging. Zh Vyssh Nerv Deiat Im I P Pavlova.

[R46] Markova EV, Obukhova LA, Kolosova NG (2003). Activity of cell immune response and open field behavior in Wistar and OXYS rats. Bull Exp Biol Med.

[R47] Markova EV, Kozlov VA, Trofimova NA, Kolosova NG (2005). Stimulation of cell component of the immune response activates exploratory behavior in senescence accelerated OXYS rats. Bull Exp Biol Med.

[R48] Sergeeva S, Bagryanskaya E, Korbolina E, Kolosova N (2006). Development of behavioral dysfunctions in accelerated-senescence OXYS rats is associated with early postnatal alterations in brain phosphate metabolism. Exp Gerontol.

[R49] Stefanova NA, Fursova AZh, Kolosova NG (2010). Behavioral Effects Induced by Mitochondria-Targeted Antioxidant SkQ1 in Wistar and Senescence-Accelerated OXYS Rats. J. Alzheimers Dis.

[R50] Stefanova NA, Fursova AZ, Sarsenbaev KN, Kolosova NG (2011). Effects of Cistanche deserticola on behavior and signs of cataract and retinopathy in senescence-accelerated OXYS rats. J. Ethnopharmacol.

[R51] Markovets AM, Saprunova VB, Zhdankina AA, Fursova AZh, Bakeeva LE, Kolosova NG (2011). Alterations of retinal pigment epithelium cause AMD-like retinopathy in senescence-accelerated, XYS rats. Aging (Albany NY).

[R52] Obukhova LA, Skulachev VP, Kolosova NG (2009). Mitochondria-targeted antioxidant SkQ1 inhibits age-dependent involution of the thymus in normal and senescence-prone rats. Aging (Albany NY).

[R53] Skulachev VP (2011). SkQ1 treatment and food restriction--two ways to retard an aging program of organisms. Aging (Albany NY).

[R54] Farr SA, Poon HF, Dogrukol-Ak D, Drake J, Banks WA, Eyerman E, Butterfield DA, Morley JE (2003). The antioxidants alpha-lipoic acid and N-acetylcysteine reverse memory impairment and brain oxidative stress in aged SAMP8 mice. J Neurochem.

[R55] Kondratov RV, Vykhovanets O, Kondratova AA, Antoch MP (2009). Antioxidant N-acetyl-L-cysteine ameliorates symptoms of premature aging associated with the deficiency of the circadian protein BMAL1. Aging (Albany NY).

[R56] Cho RH, Sieburg HB, Muller-Sieburg CE (2008). A new mechanism for the aging of hematopoietic stem cells: aging changes the clonal composition of the stem cell compartment but not individual stem cells. Blood.

[R57] De la Fuente M, Baeza I, Guayerbas N, Puerto M, Castillo C, Salazar V, Ariznavarreta C, F-Tresguerres JA (2004). Changes with ageing in several leukocyte functions of male and female rats. Biogerontology.

[R58] Shipounova IN, Svinareva DA, Petrova TV, Lyamzaev KG, Chernyak BV, Drize NI, Skulachev VP (2010). Reactive oxygen species produced in mitochondria are involved in age-dependent changes of hematopoietic and mesenchymal progenitor cells in mice. A study with the novel mitochondria-targeted antioxidant SkQ1. Mech Ageing.

[R59] Uzenbaeva LB, Vinogradova IA, Golubeva AG, Churov AV, Iliukha VA (2006). Age related changes in rat blood leucocyte count and morpho-metrical parameters of large granular lymphocytes under different light regimens. Adv Gerontol.

[R60] De la Fuente M (2010). Murine models of premature ageing for the study of diet-induced immune changes: improvement of leucocyte functions in two strains of old prematurely ageing mice by dietary supplementation with sulphur-containing antioxidants. Proc Nutr Soc.

[R61] Puerto M, Guayerbas N, Víctor V, De la Fuente M (2002). Effects of N-acetylcysteine on macrophage and lymphocyte functions in a mouse model of premature ageing. Pharmacol Biochem Behav.

[R62] Orlovskaya IA, Toporkova LB, Feofanova NA, Kolosova NG (2007). Peculiarities of bone marrow hemopoiesis in early aging OXYS rats. Bull Exp Biol Med.

[R63] Taub DD, Murphy WJ, Longo DL (2010). Rejuvenation of the aging thymus: growth hormone-mediated and ghrelin-mediated signaling pathways. Curr Opin Pharmacol.

[R64] Dorshkind K, Montecino-Rodriguez E, Signer RA (2009). The ageing immune system: is it ever too old to become young again?. Nat Rev Immunol.

[R65] Aleman A, Torres-Alemán I (2009). Circulating insulin-like growth factor I and cognitive function: neuromodulation throughout the lifespan. Prog Neurobiol.

[R66] Conrad CD, Bimonte-Nelson HA (2010). Impact of the hypothalamic-pituitary-adrenal/gonadal axes on trajectory of age-related cognitive decline. Prog Brain Res.

[R67] Li E, Kim DH, Cai M, Lee S, Kim Y, Lim E, Ryu JH, Unterman TG, Park S (2011). Hippocampus-dependent spatial learning and memory are impaired in growth hormone-deficient spontaneous dwarf rats. Endocrine Journal.

[R68] Belousova II, Gladkikh DV, Zhelezova AI, Stefanova NA, Kolosova NG, Amstislavskaia TG (2009). Age-related aspects of male reproductive function in rats with normal and accelerated senescence. Ross Fiziol Zh Im I M Sechenova.

[R69] Amstislavskaya TG, Maslova LN, Gladkikh DV, Belousova II, Stefanova NA, Kolosova NG (2010). Effects of the mitochondria-targeted antioxidant SkQ1 on sexually motivated behavior in male rats. Pharmacol Biochem Behav.

[R70] Leontieva OV, Blagosklonny MV (2011). Yeast-like chronological senescence in mammalian cells: phenomenon, mechanism and pharmacological suppression. Aging (Albany NY).

[R71] Blagosklonny MV (2006). Aging and immortality: quasi-programmed senescence and its pharmacologic inhibition. Cell Cycle.

[R72] Blagosklonny MV (2011). Progeria, rapamycin and normal aging: recent breakthrough. Aging (Albany NY).

[R73] Dazert E, Hall MN (2011). mTOR signaling in disease. Curr Opin Cell Biol.

[R74] Kapahi P, Chen D, Rogers AN, Katewa SD, Li PW, Thomas EL, Kockel L (2010). With TOR, less is more: a key role for the conserved nutrient-sensing TOR pathway in aging. Cell Metab.

[R75] Korotchkina LG, Leontieva OV, Bukreeva EI, Demidenko ZN, Gudkov AV, Blagosklonny MV (2010). The choice between p53-induced senescence and quiescence is determined in part by the mTOR pathway. Aging (Albany NY).

[R76] Leontieva OV, Blagosklonny MV (2010). DNA damaging agents and p53 do not cause senescence in quiescent cells, while consecutive re-activation of mTOR is associated with conversion to senescence. Aging (Albany NY).

[R77] Stanfel MN, Shamieh LS, Kaeberlein M, Kennedy BK (2009). The TOR pathway comes of age. Biochim Biophys Acta.

[R78] Zhao C, Vollrath D (2011). mTOR pathway activation in age-related retinal disease. Aging (Albany NY).

[R79] Maggio M, Lauretani F, Ceda GP, Bandinelli S, Ling SM, Metter EJ, Artoni A, Carassale L, Cazzato A, Ceresini G, Guralnik JM, Basaria S, Valenti G, Ferrucci L (2007). Relationship between low levels of anabolic hormones and 6-year mortality in older men: the aging in the Chianti Area (InCHIANTI) study. Arch Intern Med.

[R80] Sorwell KG, Urbanski HF (2010). Dehydroepiandrosterone and age-related cognitive decline. Age (Dordr).

